# *Synconn_build*: A python based synthetic dataset generator for testing and validating control-oriented neural networks for building dynamics prediction

**DOI:** 10.1016/j.mex.2023.102464

**Published:** 2023-10-26

**Authors:** Gaurav Chaudhary, Hicham Johra, Laurent Georges, Bjørn Austbø

**Affiliations:** a Department of Energy and Process Engineering, Norwegian University of Science and Technology (NTNU), Trondheim, Norway; b Department of the Built Environment, Aalborg University, Øst, Aalborg, Denmark

**Keywords:** Building dynamics dataset, Synthetic data, Open source, Python package, synconn_build: A python based synthetic building dynamics and operation dataset generator

## Abstract

Applying model-based predictive control in buildings requires a control-oriented model capable of learning how various control actions influence building dynamics, such as indoor air temperature and energy use. However, there is currently a shortage of empirical or synthetic datasets with the appropriate features, variability, quality and volume to properly benchmark these control-oriented models. Addressing this need, a flexible, open-source, Python-based tool, *synconn_build*, capable of generating synthetic building operation data using EnergyPlus as the main building energy simulation engine is introduced. The uniqueness of *synconn_build* lies in its capability to automate multiple aspects of the simulation process, guided by user inputs drawn from a text-based configuration file. It generates various kinds of unique random signals for control inputs, performs co-simulation to create unique occupancy schedules, and acquires weather data. Additionally, it simplifies the typically tedious and complex task of configuring EnergyPlus files with all user inputs. Unlike other synthetic datasets for building operations, *synconn_build* offers a user-friendly generator that selectively creates data based on user inputs, preventing overwhelming data overproduction. Instead of emulating the operational schedules of real buildings, *synconn_build* generates test signals with more frequent variation to cover a broader range of operating conditions.

• *Synconn_build* is an open-source tool designed to address the lack of datasets for benchmarking control-oriented building dynamics prediction models.
• The tool automates simulations, data acquisition, and EnergyPlus configuration, guided by user inputs.
• *Synconn_build* prevents data overproduction by selectively creating data, offering a user-friendly approach to dataset generation.

## Graphical abstract

**Figure:**
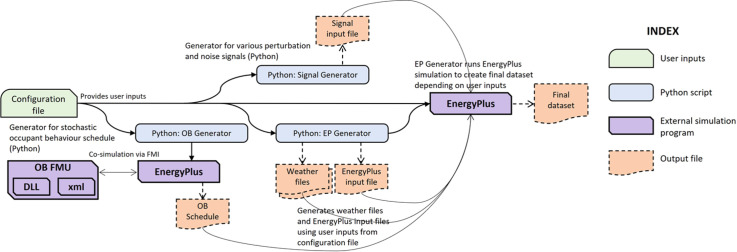

Specifications table

**Table:** 

Subject area:	Energy
More specific subject area:	Building dynamics and operation dataset
Name of your method:	*synconn_build*: A python based synthetic building dynamics and operation dataset generator
Name and reference of original method:	N.A.
Resource availability:	*synconn_build*'s codebase is hosted on a public GitHub repository (https://github.com/gaurav306/synconn_build)

## Introduction

Reducing energy demand and carbon footprint in the building sector has become a pressing concern, with buildings accounting for more than 40 % of global energy use and 36 % of greenhouse gas emissions [1]. Residential and commercial buildings consume approximately 60 % of the global electricity, with Heating, Ventilation, and Air Conditioning (HVAC) systems being responsible for almost half of this energy demand [2]. Thus, enhancing building energy efficiency while maintaining good indoor air quality has become a crucial aspect of achieving sustainability goals [3].

As buildings become larger and more multifunctional, controlling and managing various subsystems such as HVAC, lighting, power distribution, and security through Building Automation Systems (BAS) has become essential. Numerous studies have reported that a more intelligent control of these subsystems could significantly reduce energy use, leading to mitigation of greenhouse gas emissions [4]. Predictive strategies like Model Predictive Control (MPC) have been observed to be effective in reducing energy costs while maintaining comfort and considering building varying boundary conditions and internal dynamics [5,6]. The most crucial step in implementing advanced predictive control techniques in buildings is to create a control-oriented dynamic thermal model that can accurately predict changes in the indoor temperature. This model should be able to predict or calculate the effect of control actions like HVAC temperature setpoints, HVAC mode, and window opening signals on building dynamics, including indoor temperature and energy use variations. However, such a control-oriented dynamic model needs to be prompt, i.e., it should be able to predict or compute the building dynamics for the input time-period and conditions in a very short time. This is a major requirement as a predictive controller would need to call the model multiple times (typically 100 - 1000 times) depending on the search space of the control variables.

Energy models for buildings fall into three types: white-box, gray-box, and black-box [5]. White-box models(also termed as physics-based models) need detailed building and system information, using first-principle equations to calculate heat and mass transfer. EnergyPlus, TRNSYS, and IDA ICE are programs that can create such models. Gray-box models (also known as reduced-order models) utilize simplified dynamic equations fitted to data for parameter identification [7]. A common method here is Resistance-Capacitance (RC) networks, representing building elements and energy flows. Back-box models (also called data-driven models) leverage statistical or machine learning techniques, like AutoRegressive models with eXogenous inputs (ARX) and AutoRegressive Moving Average models with eXogenous inputs (ARMAX), regression trees, Support Vector Machines (SVMs), and Deep Neural Networks (DNNs), to predict energy needs and indoor conditions [8]. The different models have their own strengths and weaknesses that make them suitable for different applications. White-box models have typically been used in the design phase to ensure compliance with building energy codes, while gray-box and black-box models are more suitable for predictive control, fault detection and diagnostics during the operational phase.

Black-box models (like DNNs), while cost-effective and scalable, often need extensive training data to excel. This can be tackled using transfer learning techniques, integrating data from white-box modeling tools and system identification methods. DNNs can first be trained on a large dataset that encompasses a broad range of operating conditions and scenarios, simulated through a generic white box building model. The pre-trained network using virtual experiments can then be fine-tuned on a smaller dataset of real building operation data specific to the model predictive control problem being addressed. By leveraging the pre-trained model's ability to extract relevant features from the data, this approach can significantly reduce the time and resources required to develop effective control-oriented models, particularly in situations where the target dataset is relatively small [6,9].

Using physics-based simulation models to create datasets for pre-training of DNN models has several advantages. First, it allows for the generation of a large amount of data that covers a wide range of operating conditions and scenarios, which can improve the performance and robustness of the model. Second, it can provide a controlled environment for evaluating the performance of the model under different control strategies without the risk of damaging or disrupting the real building. Finally, it can significantly reduce the cost and time required to collect real-world data, making it a practical and scalable solution for many building control applications.

Currently, there are several public datasets available, comprising both measured data [10], [11], [12], [13] and synthetic data generated from simulations [14], [15], [16], [17]. However, it is important to consider that each dataset has its own strengths and limitations. While measurement data can provide valuable information, it is limited in evaluating control-oriented prediction models due to its restricted variation in operating conditions. The ultimate objective is to develop control-oriented black-box models using real-world building operation data. However, researchers and developers frequently encounter challenges in acquiring reliable data, which can be both empirical or simulated datasets, that have the right features, appropriate variability, and high enough quality (in terms of metadata, information gaps, and sampling rate) to properly benchmark these control-oriented models. Synthetic datasets can fill this gap, offering advantages like control over data volume and diversity, accurate ground truth, mitigation of overfitting, and privacy preservation. Such datasets can be used to pre-train, tune, evaluate, test and benchmark control-oriented black-box models over a broad spectrum of conditions. An example of a synthetic building operation dataset is AlphaBuilding [14], which includes HVAC systems, internal loads, occupant counts, environmental parameters, energy end-use, and whole-building energy demand at 10-minute intervals. However, this dataset only contains building operation data for a range of different HVAC temperature setpoints and no other possible control parameters.

Use of synthetic data to train models for real world application is not new. Simulated or synthetically generated datasets have also been used in other fields, for example, real self-driving car models pre-trained using data generated from playing driving games like Grand Theft Auto [18], learning through simulated images [19], using virtual 3D model generated image datasets to train deep neural networks for hand gesture recognition [20], and use of synthetic data for training models for healthcare applications [21].

This paper introduces a highly customizable python-based open-source tool, called *synconn_build,* that generates synthetic data for the evaluation and benchmarking of control-oriented black-box models for the prediction of building thermal dynamics. Importantly, the intention of this tool is not to generate potential schedules for real-world building operations. Rather, its primary aim is to produce a variety of test signals that challenge and test the capabilities of black-box models in understanding building dynamics, so that it is capable of handling real-world scenarios. It uses EnergyPlus, a physics-based simulation software, to perform building energy simulations. In this paper, a small 5-zone office building model serves as an exemplar, demonstrating how the tool generates synthetic data. The dataset can be generated for all 5-zones of the building for a year of simulation time with various random control features such as HVAC temperature setpoints, HVAC running mode (no operation, heating only, cooling only, or both heating and cooling), and window openings. The user can customize simulation parameters, such as simulation intervals, weather profiles, and different settings for control feature inputs. Additionally, *synconn_build* incorporates features from AlphaBuilding [14] dataset generation, such as stochastic occupancy, dynamic lighting, and Miscellaneous Electric Load (MELs) schedules. Unlike other synthetic datasets for building operations, *synconn_build* provides an easy-to-use data generator instead of generating data for all permutations and combinations of operating conditions, which can result in terabytes of data, making it difficult to use.

While this paper uses a small 5-zone office building model as a proof of concept to illustrate the capability of *synconn_build*, it is important to note that its application is not limited to this specific archetype. The tool is versatile and can be readily extended to other EnergyPlus archetype models, representing various other types of buildings. It holds the potential to generate customized datasets across a wide spectrum of building configurations, sizes, and operation conditions. A dataset generated by *synconn_build* has been used to examine the predictive capabilities of an encoder-decoder deep neural network to predict indoor air temperature evolution where control variable included heating setpoint and window opening factor [22].

In this manuscript, the Methods section first details *synconn_build*'s methodology and components, followed by the Results section, which provides examples of generated data. Information about Data Availability and Code Availability is given in subsequent sections.

## Methods

The following section details the overall workflow and different parts of the data generator used to create a synthetic dataset. The generator consists of several interconnected parts, including a stochastic occupancy schedule generator based on real occupant behavior, a random signal generator and a physics-based model of the case study building. It needs to be stressed that while a small 5-zone office building model is utilized as a proof of concept and case study building in this paper, the tool's utility is not confined to this archetype, but extends to a diverse range of EnergyPlus models, demonstrating its broad versatility in handling different building types and configurations.

### Workflow and usage

**Fig. 1:**
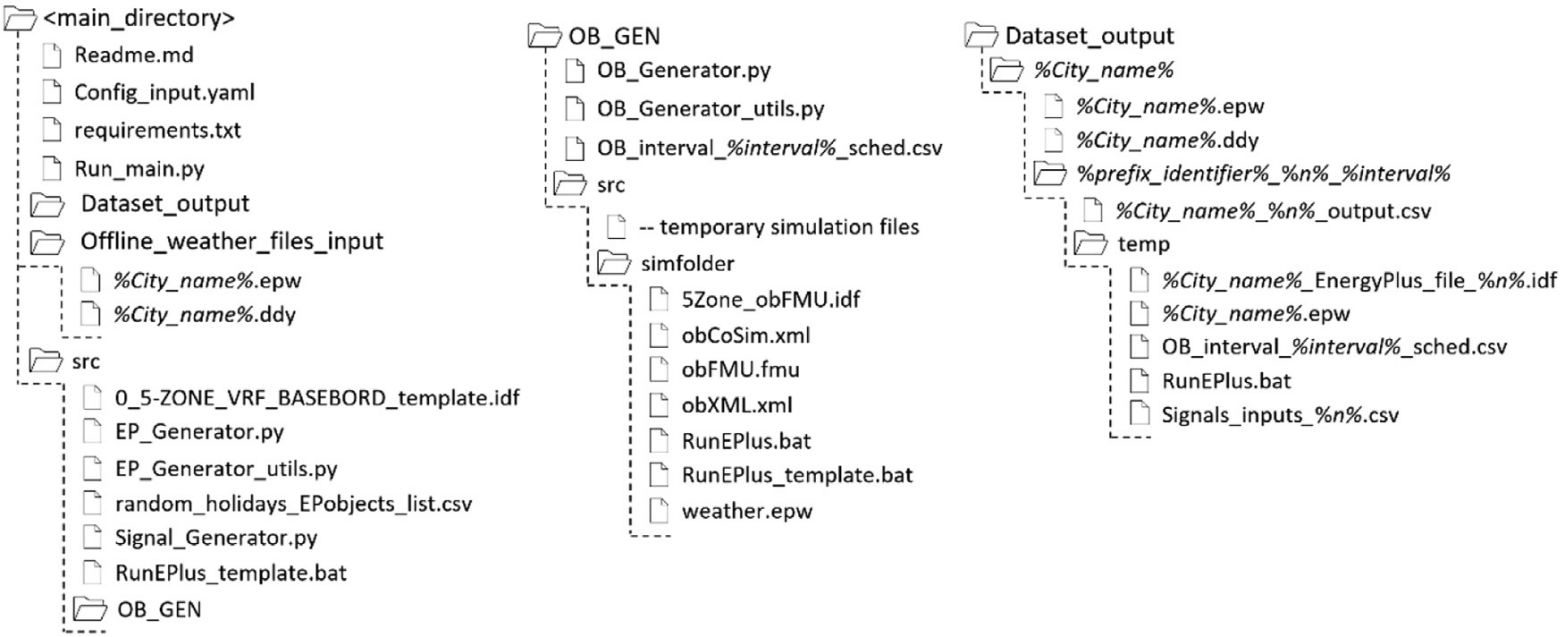
File structure of the codebase for *synconn_build*.

The codebase for the data generator comprises three main folders, as shown in Fig. 1: ‘*Dataset_output/’*, ‘*Offline_weather_files_input/’*, and ‘*src/’*. The ‘*Dataset_output/’* folder contains the final time series data generated, which is further described in the 'Data availability' section. The ‘*src/’* folder is dedicated to all the scripts and auxiliary files required by the data generator. As discussed before, *synconn_build* enables the user to create customized datasets for a variety of settings. These options are summarized in the ‘*Config_input.yaml’* configuration file, in which users can modify various variables according to the instructions provided in the comments. This configuration file is critical to the *synconn_build* 's functionality, as all the scripts depend on its inputs.

**Fig. 2:**
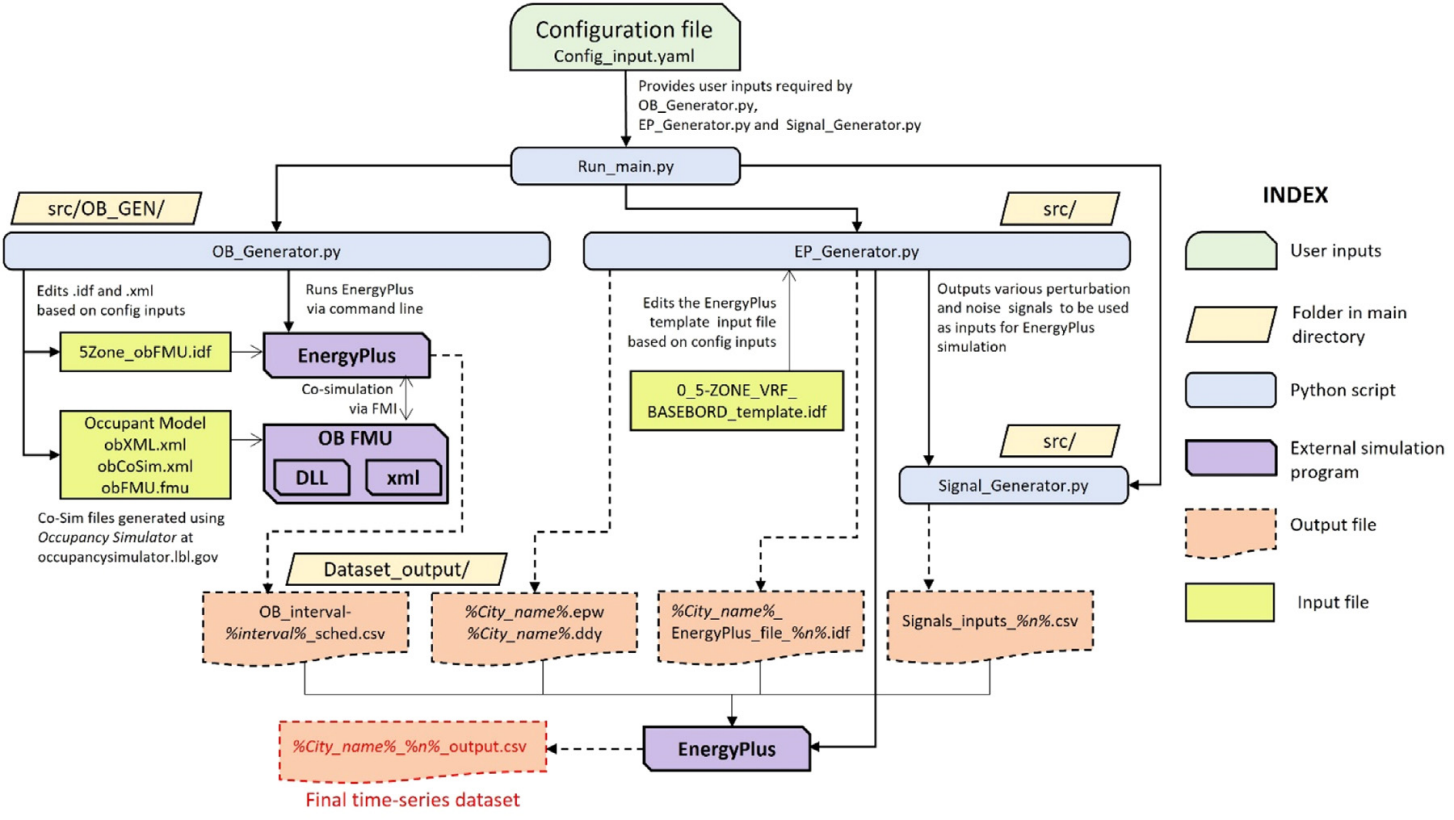
High-level workflow of *synconn_build*.

The ‘*Run_main.py’* script triggers the data creation pipeline, depicted in Fig. 2, which provides a high-level overview of *synconn_build* 's workflow. This script transforms the text data from ‘*Config_input.yaml’* into a dictionary that serves as the settings for the entire pipeline. The main script calls ‘*src/OB_GEN/OB_generator.py’* to generate stochastic occupancy schedules depending on user settings. It then calls ‘*src/Signal_generator.py’* to generate different random signals used as schedules in the EnergyPlus simulation. Finally, it calls ‘*src/EP_Generator.py’*, which handles the complex task of manipulating and editing the EnergyPlus Input Data File (IDF). It also downloads weather files, creates the necessary directories, and finally initiates the EnergyPlus program to perform the simulations. The components of this pipeline will be discussed in greater detail in the subsequent sub-sections.

### Occupant behavior (OB) schedule generator

*Synconn_build* generates stochastic occupancy schedules for all the zones in the building to be simulated using the OB modeling tool [23]. The latter tool uses an occupant behavior Functional Mock-up Unit (*obFMU*) that enables co-simulation with EnergyPlus implementing a Functional Mock-up Interface (FMI). The co-simulation with EnergyPlus requires the *obFMU.fmu* file, which in turn requires an *.xml* file based on an *obXML* (occupant behavior eXtensible Markup Language) schema and a co-simulation configuration file. All the required files for co-simulation can be generated using the app hosted at https://occupancysimulator.lbl.gov/. This app takes high-level input on occupants, spaces and events to generate either simulated occupant movement and schedules for each space or the co-simulation files, which can be used for simulation externally. The advantage of using the co-simulation files is that the different occupant schedules for each space can be generated repeatedly offline. Because of the stochastic nature of the co-simulation, the schedules generated will vary each time the co-simulation is run. This flexibility allows the generation of more time-series datasets with the same boundary conditions.

The script ‘*src/OB_GEN/OB_Generator.py’* manages the EnergyPlus-obFMU co-simulation to generate stochastic occupancy schedule files. The output occupancy files (in the form of text CSV files) are stored in the ‘*src/OB_GEN/’* folder (see Fig. 2). The user can choose the time interval of the occupancy schedules by changing the appropriate setting in the configuration file. Further step-by-step instructions on how to use it are given in the "*Readme.md*" file provided in the main directory of the codebase. A high-level workflow for occupancy schedule generation can be found in Fig. 2.

### Signal generator

In order to design a data-driven control-oriented model that is capable of predicting a building's response to a set of control variables, the model needs to train on data representing the potential variation in these variables. One approach is to generate distinct datasets for each unique combination of control variables. However, the sheer number of combinations, influenced by the number of control variables and their potential values, can lead to an immensely large dataset. Alternatively, a dataset can be developed wherein control variables fluctuate randomly. This technique of utilizing random signals is a cornerstone in the field of system identification [7]. In this context, perturbation signals are specifically introduced to gage and analyze the system's response. By employing random signals across all control variables, system dynamics can be captured similarly to the methodical exploration of each control variable combination in the former approach. It is recommended to use signals that have no correlation with other inputs and approximate white noise properties [7]. Varying the frequency of change for a signal, i.e., how often the state of the signal changes, can help identifying different time constants of a system. However, a signal with a mix of frequencies can help capture more time constants. Signal generation is implemented in the 'src/Signal_Generator.py' script. This script allows the user to generate three distinct types of random perturbation signals individually for all zones of the case study building:

(1) *HVAC setpoints* (termed as "*mprs_setpoints*" in the configuration file): this signal generates a multi-level random signal for the heating setpoint of HVAC system in all five zones. The cooling setpoint is defined as the heating setpoint plus 5 °C for every zone. The difference between the heating and cooling set point can be configured by the user. The user can define a set of possible heating setpoints from which the signal generator chooses randomly, for example, [18 °C, 19 °C, 20 °C, 21 °C, 22 °C]. The user can also define the way random selections are made, selecting between uniform distribution or normal distribution.
(2) *HVAC mode* (termed as "*mprs_hvac_mode*" in the configuration file): this signal generates a multi-level random signal for the 4 different HVAC mode individually in all five zones. These modes are as follows:
  • 0 – uncontrolled/free floating: in this mode, neither heating nor cooling is enabled.
  • 1 – only heating is allowed if required.
  • 2 – only cooling is allowed if required.
  • 3 – both heating and cooling are enabled if required.
(3) *Window opening*: this signal type allows the user to control how the windows are opened and closed. The user can configure a set of possible window positions from 0 to 1, for example, [0, 0.25, 0.5, 0.75, 1], where 0 is fully close and 1 is fully open. The user can also configure the lower and upper limit for the number of timesteps the window can be open (before reverting to 0). This signal is individually generated for all the windows in the building.

For all the generated signals, the user can adjust the frequency of change. This frequency of change can be configured by limiting the range of possible timesteps for the signal, by individually configuring five different possibilities for the level of frequency of change.

Furthermore, the user can choose to select the signal to have just one level of frequency of change or make a mix of levels. The mix of levels is generated by splitting the length of the required signal in a random number of randomly long chunks and then designating each chunk a randomly selected level of frequency change. Detailed instructions on how to configure these features can be found in the *Readme.md* file in the Github repository.

### Noise signals

When using advanced and intricate data-driven models such as DNNs, having a small dataset can result in poor performance and overfitting. This is because small datasets may pose a more challenging mapping problem for neural networks to learn [24]. A small dataset can either have a limited number of data points or sparsely sampled points in the high-dimensional input space. Adding random noise during the training of a neural network model is an effective technique for reducing the generalization error and enhancing the structure of the mapping problem [25,26].

**Fig. 3:**
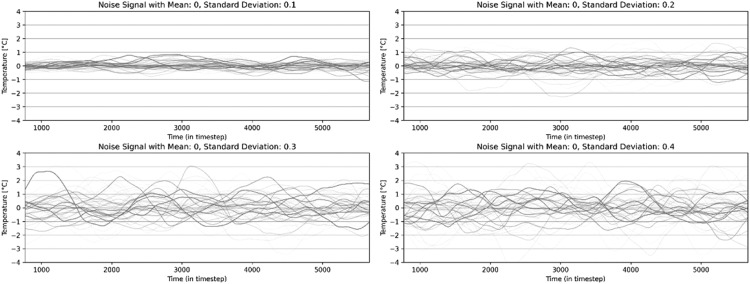
Noise signals for HVAC setpoints.

In *synconn_build*, random noise was added to HVAC temperature setpoints in the EnergyPlus simulation in addition to HVAC temperature setpoints signal. The noise was generated by smoothening the Gaussian noise with user-defined mean and standard deviation [27]. This kind of noise would simulate HVAC system-based malfunctions, faults, mismatch of temperature setpoints and measurement errors from temperature sensors. Fig. 3 shows 50 random noise signals with a mean of 0 °C and four different standard deviations.

### Modeling assumptions

**Fig. 4:**
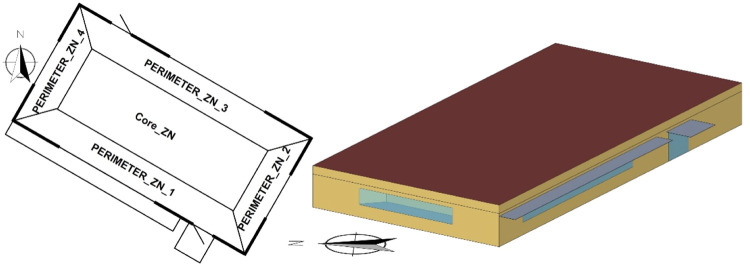
Thermal zones and building geometry of the case study building used for this paper.

*Synconn_build* employs the building energy simulation program EnergyPlus v22.1.0 [28] to generate building data. The US Department of Energy has created a collection of reference EnergyPlus models for commercial buildings that represent 70 % of such buildings in the US [29]. These models have been used in various applications. The specific model used in *synconn_build* is a small office building with a single floor and dimensions of 30 m x 15 m and a ceiling height of 2.4 m, oriented 30° East of the North direction. A 3D visualization and zone configuration can be seen in Fig. 4. The building comprises four exterior thermal zones and one interior thermal zone, with two meeting rooms (PERIMETER_ZN_2 and PERIMETER_ZN_3) and three open offices. The building has one window on each of the four facades and glass doors on the southwest and northeast facades, with overhangs providing shading for the southwest facing window and door. There are no internal openings between zones. The U-values of the internal and external walls are 1.6 W/m²K and 2.8 W/m²K, respectively.

All fenestrations are high-performance windows with a U-value of 0.7 W/m²K. The doors in PERIMETER_ZN_1 and PERIMETER_ZN_3 is single pane glass with U-value of 5.8 W/m²K. The window-to-wall ratio is approximately 0.29. To reduce overheating and simulate occupant preferences for low glare, automatic window shading control lowers the interior shade when the solar irradiance on windows exceeds 50 W/m^2^. To simulate the opening and closing of the windows, the *ZoneVentilation:WindandStackOpenArea* EnergyPlus object [30] is employed. Using this object, the opening and closing of windows can be scheduled via an EnergyPlus schedule connected to an external CSV file. The equation used to calculate the wind-driven natural ventilation rate is based on the "Wind and Stack with Open Area" model in EnergyPlus.

The building uses a Variable Refrigerant Flow (VRF) HVAC system [31] for conditioning of the zones. A VRF HVAC system typically consists of an outdoor heat pump and several indoor air terminal units. The outdoor heat pump is used to cool and heat the refrigerant sent to all the individual air terminal units. For this model, an air-to-air heat pump supplies the refrigerant for air terminals in the interior zone. A water-to-air heat pump served by a condenser loop with an electric boiler and cooling tower is used for exterior zones. The air terminals in each zone have a heating and cooling coil for heating and cooling using the same terminal. The ventilated air for the building is delivered by a dedicated outdoor air system. For this case study, a timestep of 15 min was used for the simulation. The user can control this parameter using the *Config_input.yaml* file

The temperature setpoints for the HVAC systems are provided by the signal generator described hereafter. The signal generator script primarily generates the heating setpoint. The setback temperature setpoint, i.e., temperature setpoint during unoccupied hours, for both heating and cooling was different for all zones. It varied as ±1 °C, ±1.5 °C, ±2 °C, ±2.5 °C and ±3 °C respectively for five zones. For example, for ZN_1 the setback temperature for cooling was set to the cooling setpoint +1 °C and setback temperature for heating was set to the heating setpoint −1 °C. Different setbacks were used to create further variation in HVAC temperature setpoints profiles for all five zones. This is configured inside the EnergyPlus IDF file.

**Fig. 5:**
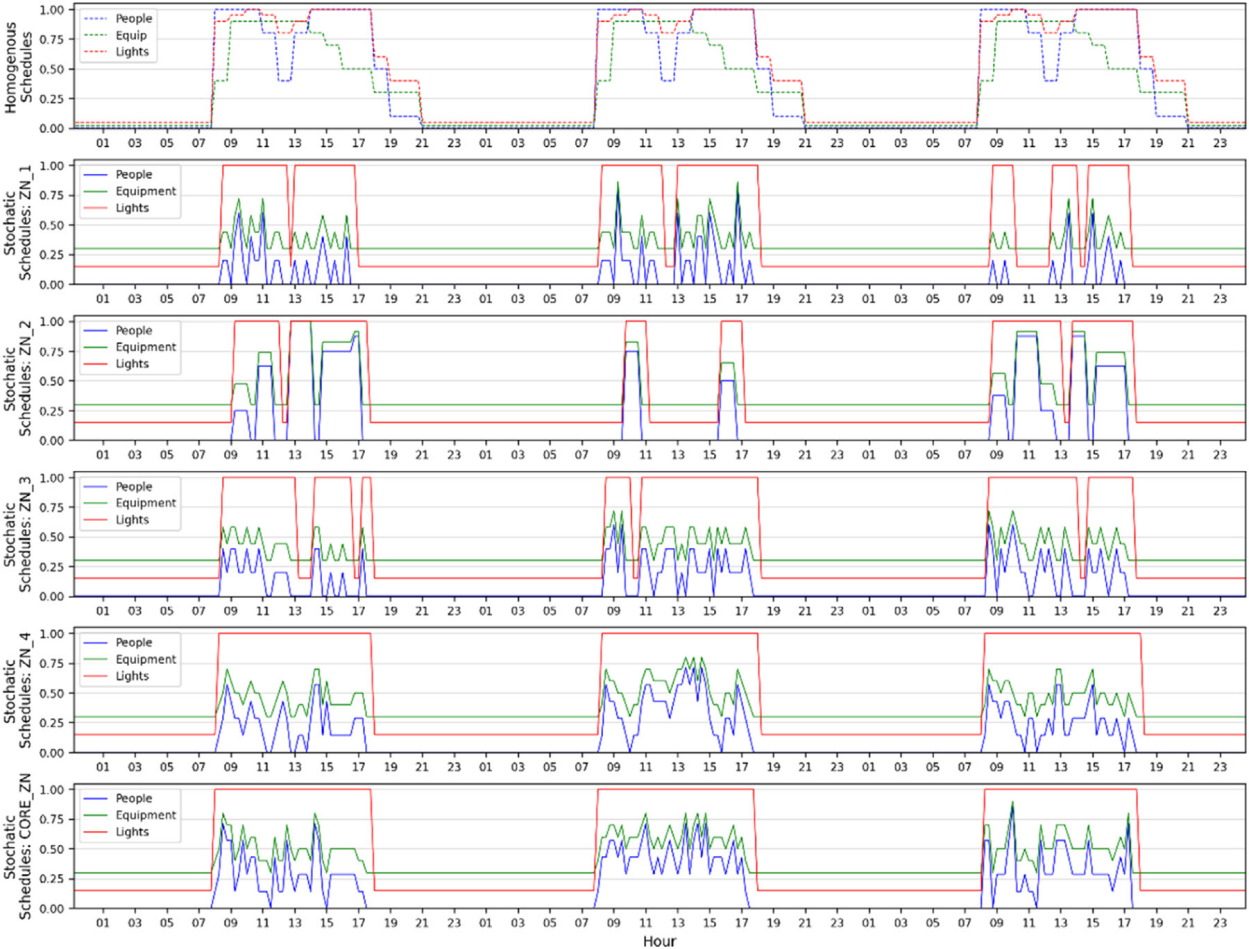
Stochastic occupancy, lighting and MELs schedules for five zones compared to homogenous schedules.

**Fig. 6:**
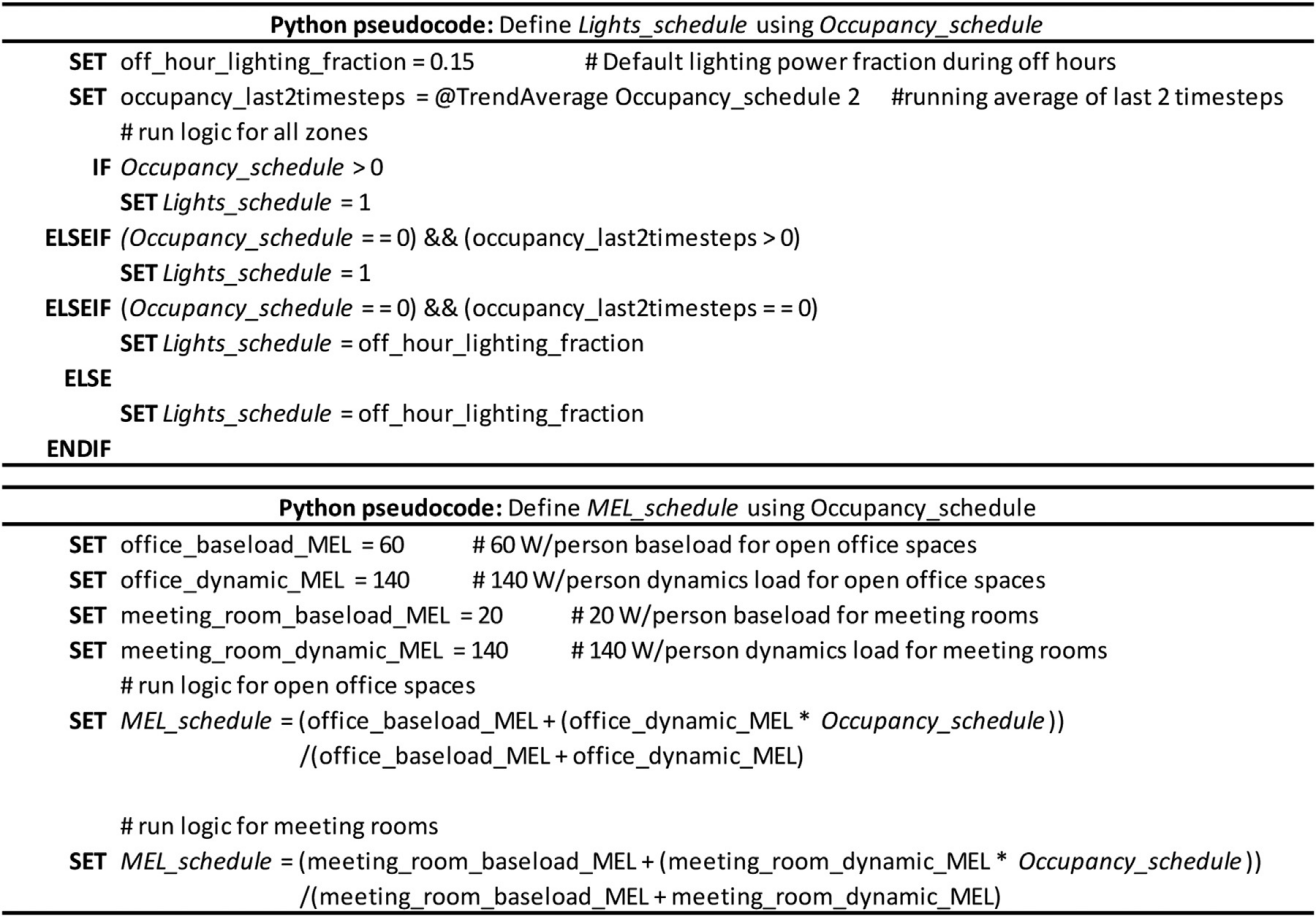
EnergyPlus Runtime Language (ERL) pseudocode for defining lighting and miscellaneous electric loads (MELs) schedules.

The schedules for occupancy were produced using a stochastic occupancy simulator [23] to represent the random movements of occupants in an office building. These schedules thus encompass spatial and temporal variations of real-life occupancy in an office building, which is very different from homogenous and static occupant schedules that are very often used in building simulations. In various studies, lighting and MELs have been found to have a direct correlation with occupancy [23,32,33]. Based on the latter and using the methodology presented by Hong et al. [15], the lighting and MELs schedules were implemented in EnergyPlus using Energy Management Script (EMS) programs. The schedules for three working days of occupancy, lighting and MELs are presented in Fig. 5, together with standard regular occupancy schedules. The logic used for the lighting and MELs schedules is described as a pseudocode in Fig. 6.

### Weather files

EnergyPlus requires weather data in the form of *.epw* files for simulation. To obtain this data, users have the option of providing a download link to weather data from https://climate.onebuilding.org/ (a website with a vast collection of free-to-use climate data for building performance simulation programs) or providing *.epw* and *.ddy* files. The ‘src/*EP_Generator.py’* script uses *.epw* file as weather file for EnergyPlus simulation and *.ddy* file to add sizing design day objects to the EnergyPlus input file.

### Random number generator

*Synconn_build* uses the *numpy.random* python library, which employs PCG-64 as its underlying random number generator, to generate pseudorandom numbers. PCG-64 is an implementation of O'Neill's permutation congruential generator with a 128-bit architecture [34].

## Results

In this section, examples of the generated data are showcased to emphasize the utility and flexibility of the tool. After examining the Methods section, readers can visualize the diverse scenarios that *synconn_build* can generate, encompassing varied control variables settings and output time-series data. It is to be noted that the results here are shown for one zone(PERIMETER_ZN_1) of the case study building and not the whole building.

### Signal generation

**Fig. 7:**
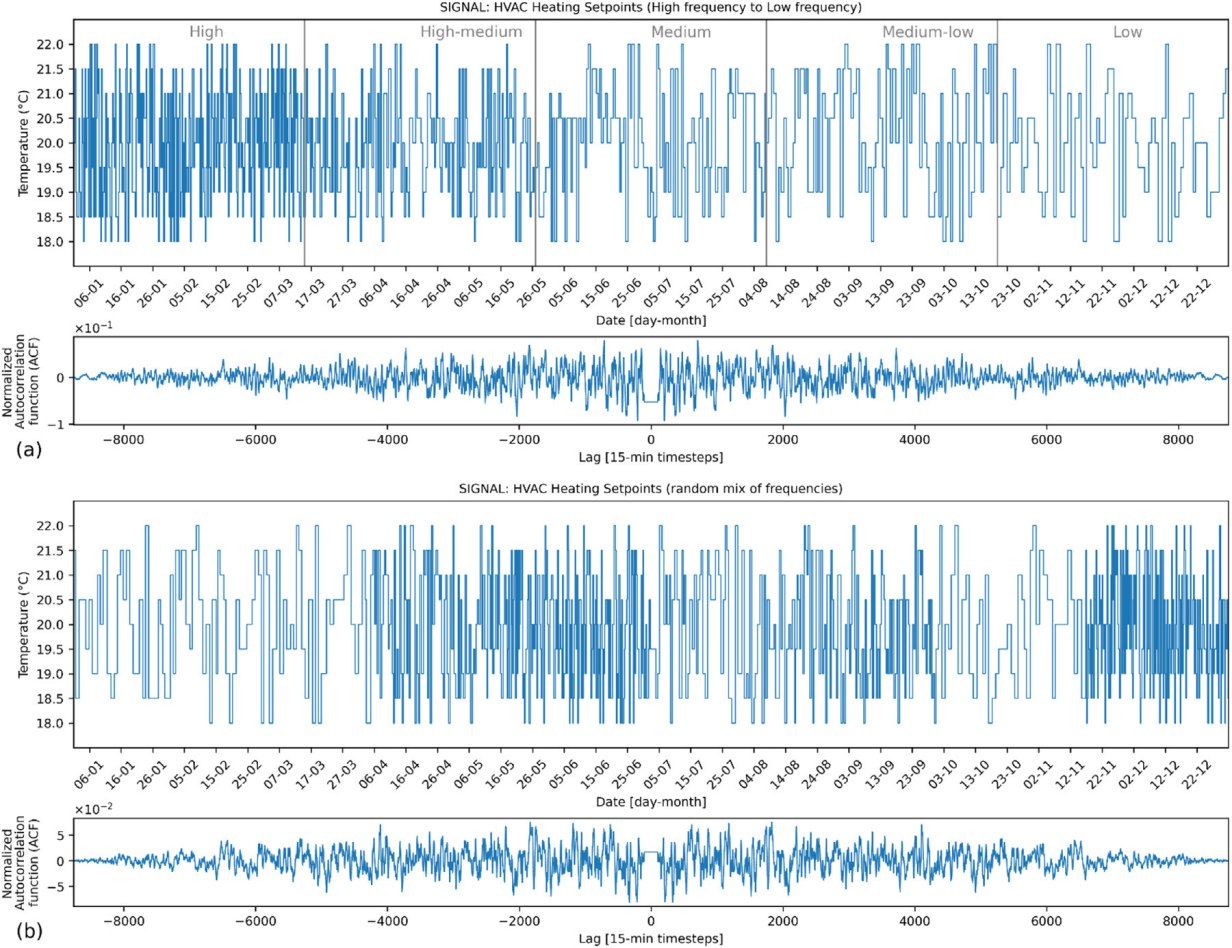
Example HVAC heating setpoint signal.

**Fig. 8:**
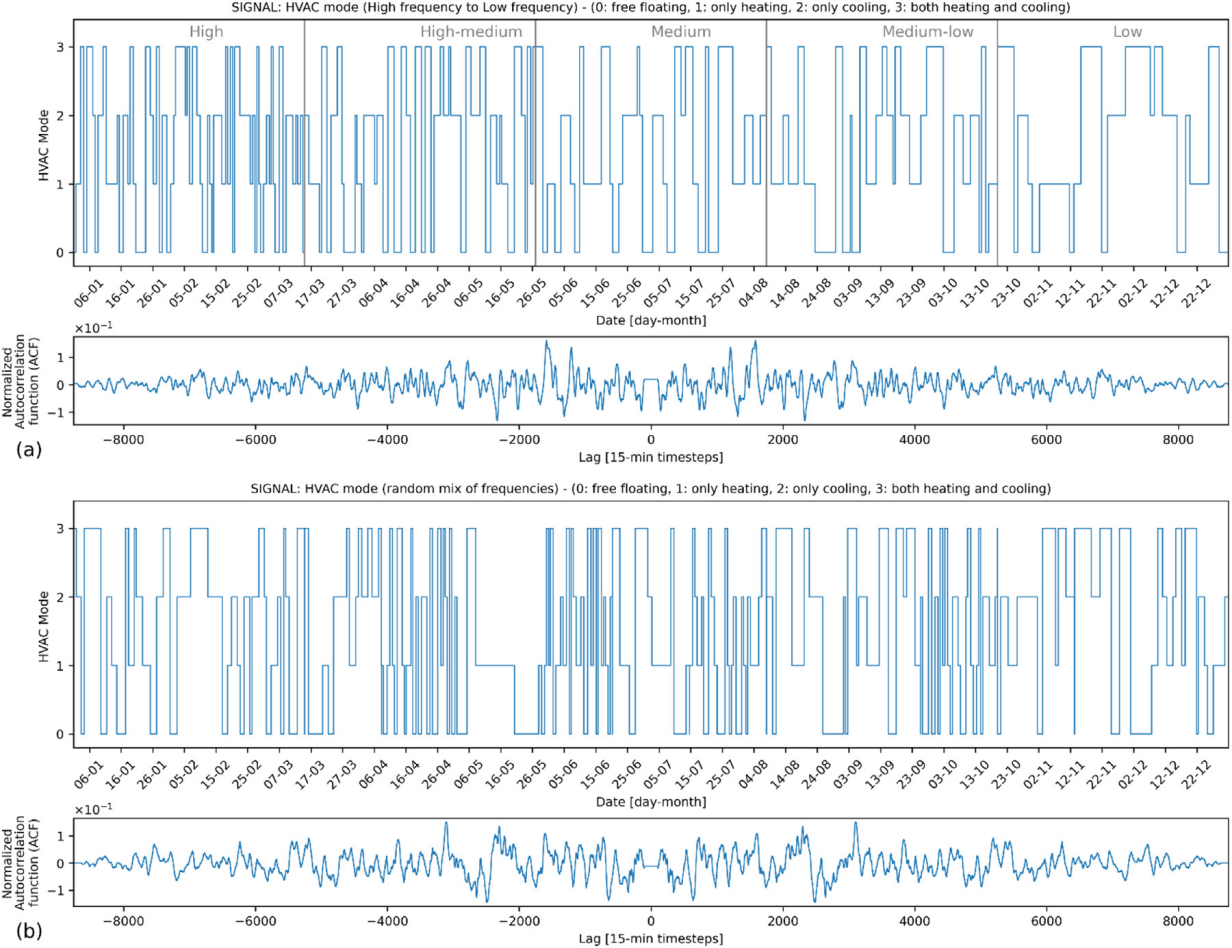
Example HVAC running mode signal.

**Fig. 9:**
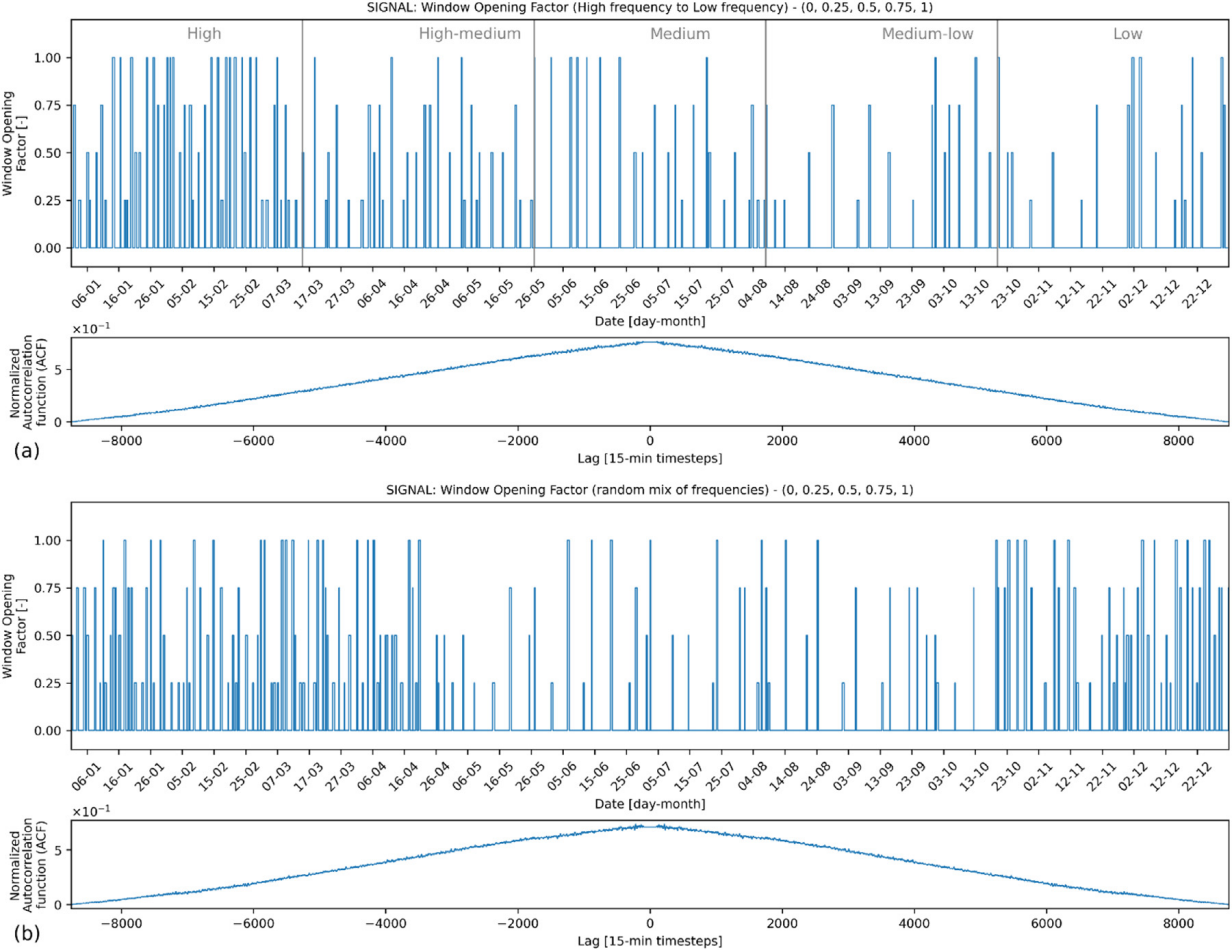
Example Window opening factor signal.

Fig. 7, Fig. 8, Fig. 9 present examples for HVAC heating setpoint, HVAC running mode and window opening, respectively, for a yearlong energy simulation generated by *synconn_build*. In each of the figures, the plot for the signal is followed by a normalized self-correlation coefficient plotted against the lag of the signal relative to the signal itself (generated by the *scipy.signal.correlate* function in Python). The x-axis of this plot represents the lag, varying from −8760 to +8760. It corresponds to the number of hours in a year, i.e., length of the signal. Positive lags mean that the second signal is shifted to the right of the first signal, and negative lags mean that it is shifted to the left. The self-correlation coefficient usually has a large central spike at 0 lag, which indicates that the signal is perfectly correlated with itself when there is no shift. Here, the central spike is removed to make it easier to see coefficient values for other lags. This is done by removing all the values for lags between −96 and +96. For all other lag values, the self-correlation is close to zero and without any spikes, indicating that the signal is random without specific periodicity. Consequently, there is no discernible pattern in the time series and one observation cannot be used to predict the next ones, which is a characteristic of white noise.

Subplot (a) in Fig. 7, Fig. 8, Fig. 9 shows a full year split in 5 equal-sized sections with the frequency of change ranging from high to low. Subplot (b) shows a full year with a random mix of frequencies. Fig. 7 displays the heating setpoint signal, illustrating random values within the set that range from 18 °C to 22 °C, incremented by 0.5 °C intervals. Fig. 8 shows the HVAC mode with possible values in set [0,1,2,3]. The Fig. 9 shows the signal for window opening factor with possible values in set [0,0.25,0.5,0.75,1]. A non-uniform distribution is used for the window opening signal, with a higher likelihood of the value being 0. The other values are equally likely. When 0 is not included in the set, the distribution is fully uniform. The observed triangular shape in the autocorrelation function corroborates this non-uniform distribution. As the lag deviates from 0 in either direction, the autocorrelation decreases, reflecting a diminished likelihood of observing the same value, especially given the dominance of the value 0. Essentially, repeated occurrences of the value 0 lead to sequences of similar values, causing positive autocorrelations for short lags. With increased lags, the chances of observing the same value decline, aligning with the observed decrease in autocorrelation values farther from lag 0.

The settings for various levels of frequency of change can be modified by the user using the *Config_input.yaml* file to have more control over the time dynamics that need to be captured.

### Random HVAC heating and cooling setpoints

**Fig. 10:**
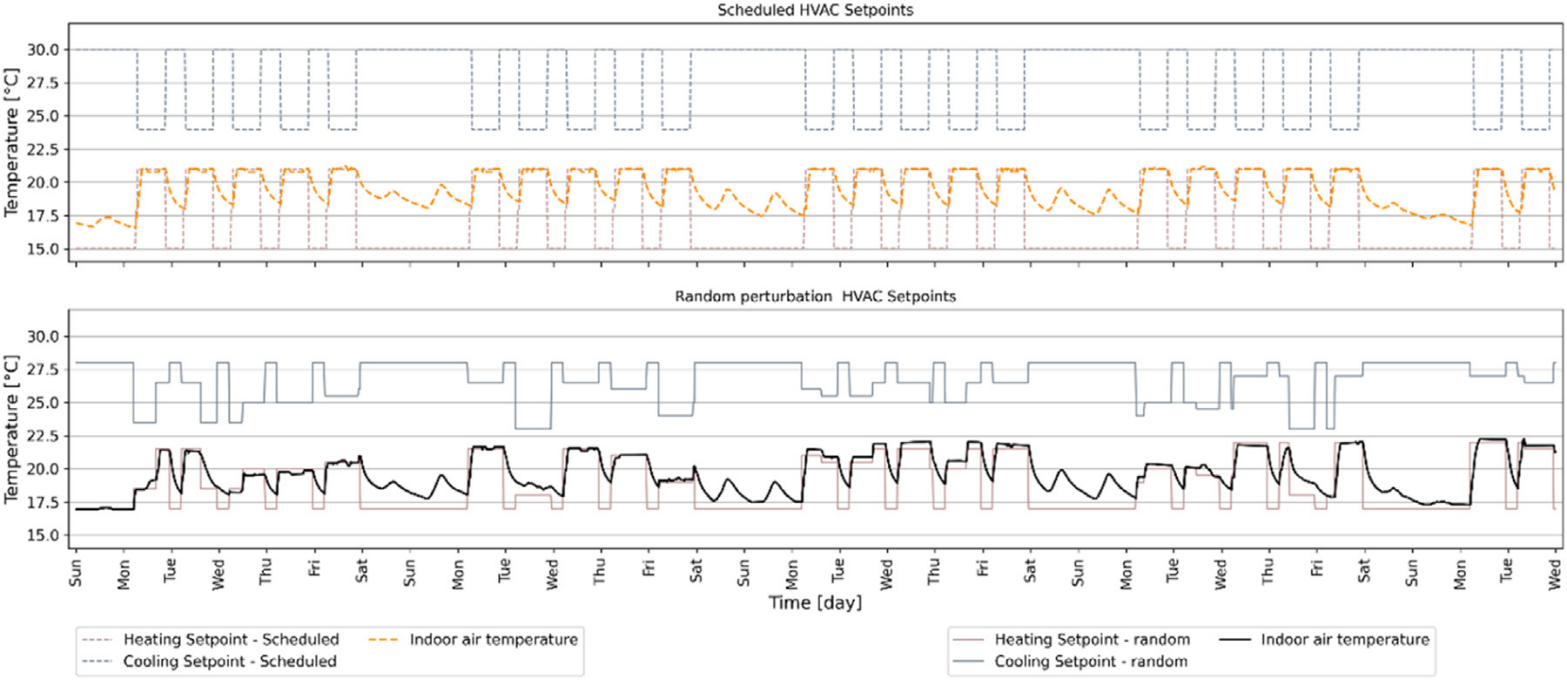
Examples of heating and cooling temperature setpoint signal.

**Fig. 11:**
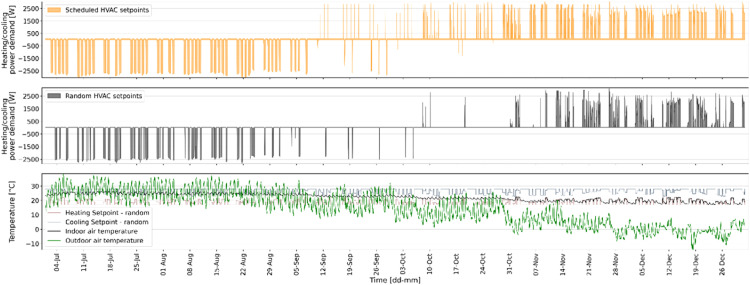
Example of how heating and cooling power demand would be different for random vs schedules HVAC setpoints.

For traditional building energy simulations, typically "Scheduled setpoints" are used for heating and cooling setpoints. For example, the heating setpoint might be set to 21 °C and the cooling setpoint to 24 °C for occupied hours, with setback temperatures during unoccupied hours. Fig. 10 depicts the difference in IAT evolution between random and scheduled sets of heating/cooling setpoint during a heating-dominated 31-day period. Fig. 11 shows the heating and cooling power demand during a six-month period. Heating demand is shown as positive values and cooling demand is shown as negative values. Scheduled temperature setpoints lead to repetitive IAT evolution and power demand. Although using scheduled temperatures can aid a data-driven model in recognizing building dynamics for a specific set of temperature values, it may not be conducive to learn how the building dynamics will change with different temperature setpoints. The random setpoint signals allow the data-driven model to learn how the building dynamics will evolve when HVAC setpoint is changed from one value to another.

### Random HVAC running mode

**Fig. 12:**
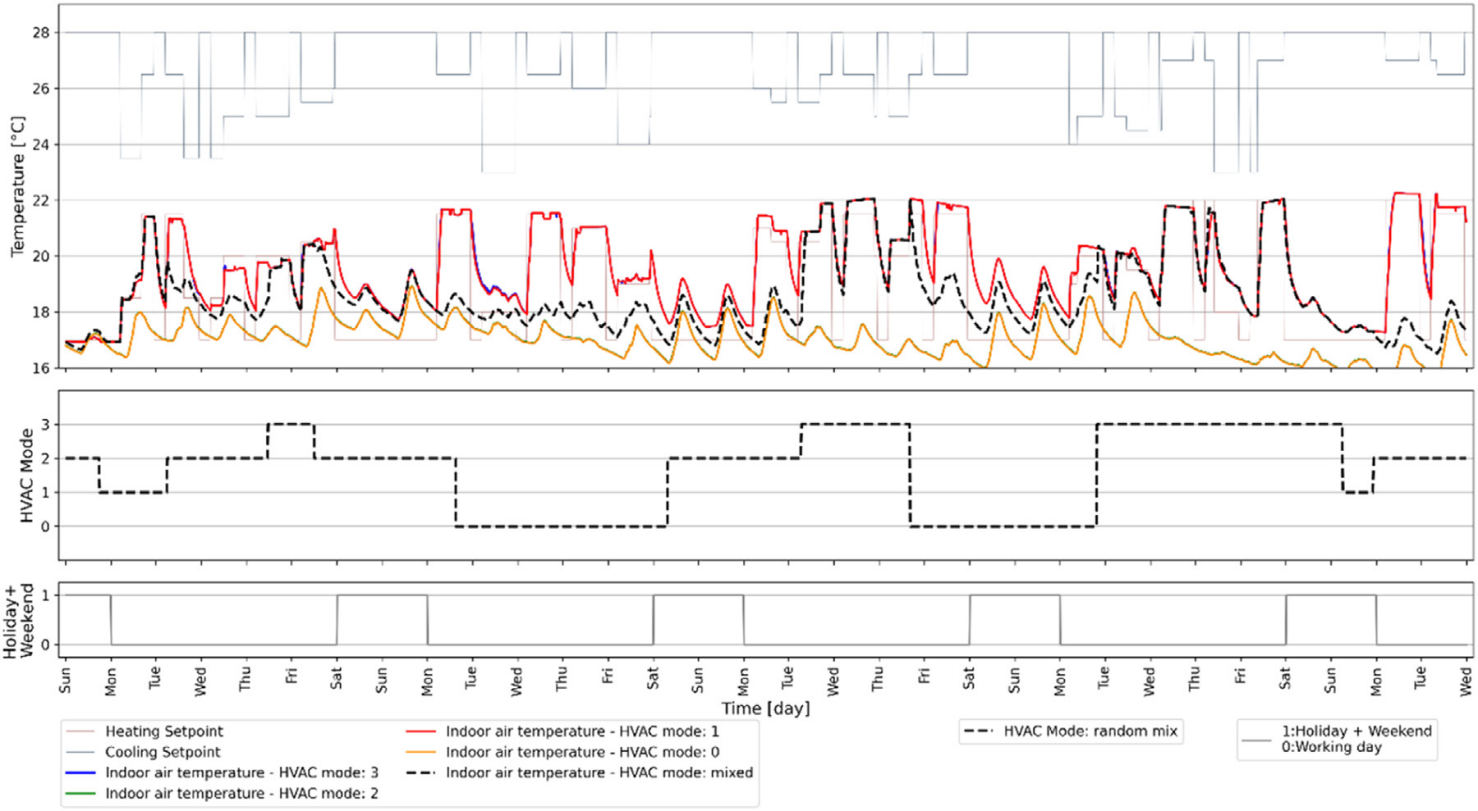
Example of how HVAC mode would affect the indoor air temperature.

**Fig. 13:**
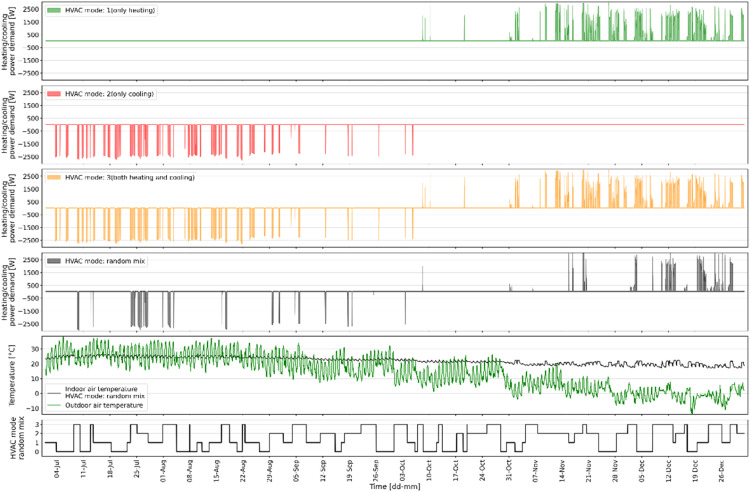
Example of how HVAC mode would affect the heating and cooling demand.

A predictive controller can assess whether the energy demand can be decreased by controlling the HVAC system using prediction models that have learned the building dynamics when heating and/or cooling is enabled. In order to develop a data-driven model for this purpose, the HVAC system in EnergyPlus building model was programmed to operate randomly in four distinct modes as mentioned in the Methods section. Fig. 12 presents the varying IAT under different HVAC modes during the same time frame and boundary conditions as displayed in Fig. 10. The first subplot displays the IAT for different HVAC modes that are either fixed for the entire 31-day period or varying randomly, given by the random HVAC mode schedule in the second subplot. The third subplot shows a holiday schedule with working days (designated by 1) and holidays/weekends (designated by 0). Fig. 13 demonstrates the energy use of the VRF system for different HVAC modes, i.e., mode 0, 1, 2 or 3, and a random mix of all four, during the same time frame and boundary conditions as illustrated in Fig. 11 for the same 31-day period.

The results in Figs. 12 and 13 indicate that by using just one signal with a random mix of HVAC running modes, the building dynamics of all modes and the impacts of different boundary conditions can be captured.

### Random window opening

**Fig. 14:**
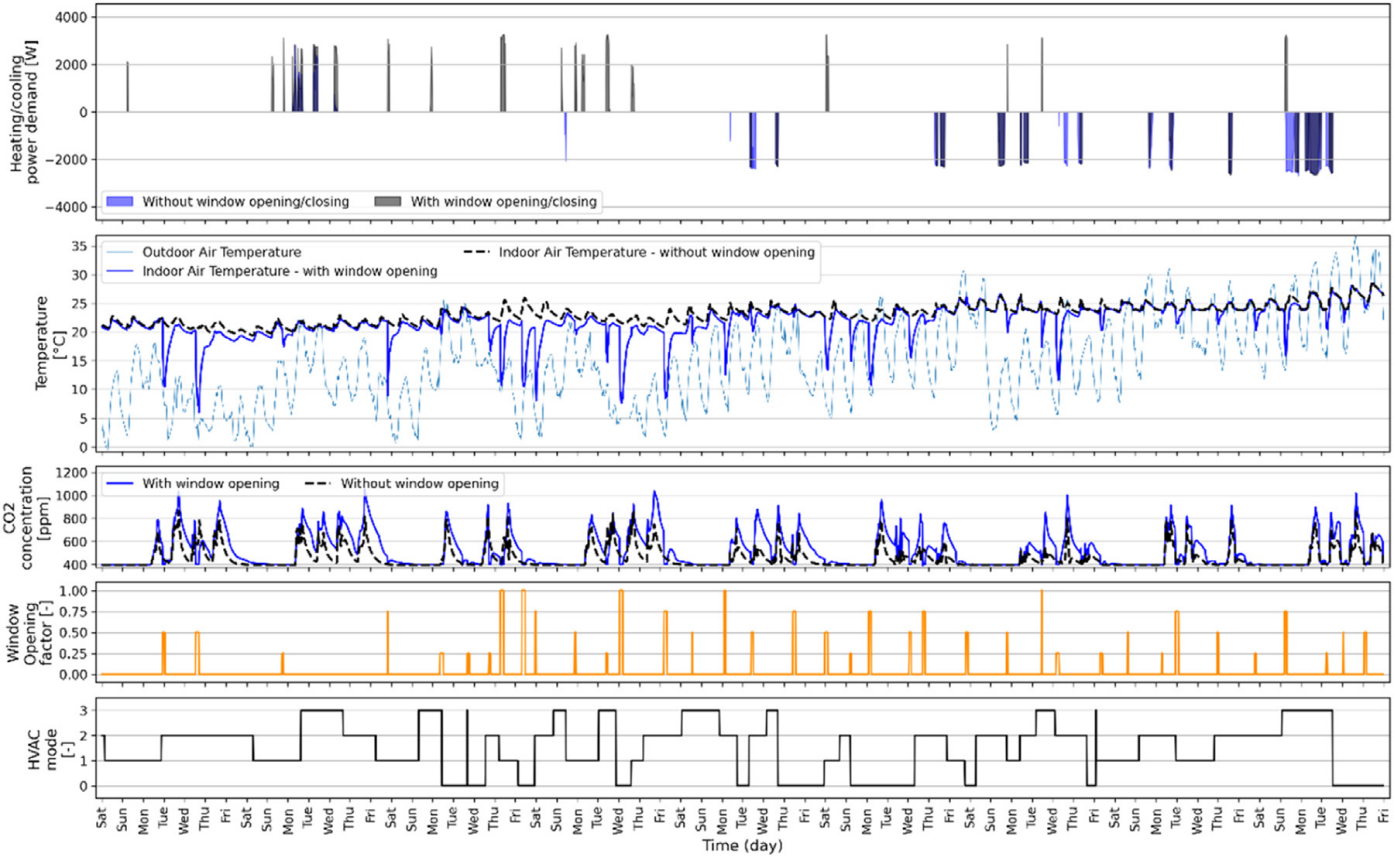
Example of how window opening would affect the indoor air temperature.

The purpose of the random window opening signal is to explore how a data-driven model can predict the impact of opening a window on the dynamics of a building. This impact depends on the temperature difference between indoor and outdoor environments and the mode of the HVAC system. When a window is opened or closed, the IAT experiences a sudden change. It then gradually stabilizes once the window signal is reversed. The opening of a window also affects the CO_2_ concentration in the zone and the energy use of the HVAC system. By using a dataset generated by *synconn_build*, a trained model should be able to leverage window opening as a means to enhance comfort and reduce energy needs. Fig. 14 illustrates a 60-day simulation period with both random HVAC mode and random window opening signals. The first, second and third subplot shows the energy use, sudden changes in IAT and CO_2_ concentration, respectively, whenever the window is opened and closed for certain HVAC mode. The fourth subplot shows the window opening signal, where 0 corresponds to the window being closed and 0.25, 0.5, 0.75, and 1 indicates the relative effective window opening area. In this case, the window remains open from 2 to 16 timesteps (i.e., 30 min to 4 h). The fifth subplot shows the random HVAC mode schedule used here. The aim is to capture building dynamics for varying HVAC mode and opening/closing of window during different boundary condition using just one simulation, resulting in less generated data. The choice of a 60-day simulation period was specifically made to encompass scenarios where the outside air temperature is both higher and lower than the IAT. This was driven by the goal to demonstrate diverse spikes in indoor air temperature (IAT) and subsequent variations in both heating and cooling demand. By showcasing such periods, it illustrates the range of potential effects on power demand and temperature fluctuations.

## Data structure

This section provides the structure of the dataset, and the way data is saved in different directories.

### Time-series data

The time-series dataset generated by the EnergyPlus simulation engine is provided in CSV file format. The first column is labelled *"Date/Time"*, representing the date and time of each timestep, while the remaining columns correspond to different variables and outputs related to building dynamics (as summarized in Table 1). The generated time series dataset can be conveniently analysed using any programming tool or software for CSV files.

**Table 1:** Final time-series dataset file summary.

Column #	Category	Column name	Unit	Comments
1	Date/Time	Date/Time	[-]	Format: *%d/%m%H:%M:%S*
2	Weather	E nvironment:Site Outdoor Air Drybulb Temperature [C](TimeStep)	°C	
3		Environment:Site Outdoor Air Relative Humidity [%](TimeStep)	%	
4		Environment:Site Wind Speed [m/s](TimeStep)	m/s	
5		Environment:Site Diffuse Solar Radiation Rate per Area [W/m2](TimeStep)	W/m^2^	
6		Environment:Site Direct Solar Radiation Rate per Area [W/m2](TimeStep)	W/m^2^	
7–11	Zone Internal Loads	*%zone_name%*:Zone People Occupant Count [](TimeStep)	[-]	5 columns for each with*%zone_name%* = [PERIMETER_ZN_1, PERIMETER_ZN_2, PERIMETER_ZN_3, PERIMETER_ZN_4, CORE_ZN]
12–16		*%zone_name%*:Zone Lights Electricity Rate [W](TimeStep)	W	
17–21		*%zone_name%*:Zone Electric Equipment Electricity Rate [W](TimeStep)	W	
22	Holiday	EMS:Holiday [](TimeStep)	[-]	
23	Occupied	EMS: Occupied [](TimeStep)	[-]	
24–28	Window Opening Factor	EMS:WINDOW_*%zone_name%*_OPENING_FACTOR [](TimeStep)	[-]	5 columns for each with%*zone_name%* = [PERIMETER_ZN_1, PERIMETER_ZN_2, PERIMETER_ZN_3, PERIMETER_ZN_4, CORE_ZN]
29–33	HVAC Operation Mode	EMS:HVAC_*%zone_name%*_OPERATION_MODE [](TimeStep)	[-]	
34–38	HVAC Heating Setpoint	E MS:*%zone_name%*_HEATING_SETPOINT [](TimeStep)	°C	
39–43	HVAC Cooling Setpoint	E MS:*%zone_name%*_COOLING_SETPOINT [](TimeStep)	°C	
44–48	Zone Indoor Air Temperature	E MS:*%zone_name%*_INDOOR_AIR_TEMPERATURE [](TimeStep)	°C	
49–53	Zone VRF Heating Energy Use	EMS:VRF_HEATING_POWER_DEMAND_*%zone_name%* [](TimeStep)	W	
54–58	Zone VRF Cooling Energy Use	EMS:VRF_COOLING_ POWER_DEMAND _*%zone_name%* [](TimeStep)	W	
59–63	Zone All HVAC Energy Use	EMS:ZONE_HVAC_ POWER_DEMAND _*%zone_name%* [](TimeStep)	kW	
64–69	Zone CO_2_ concentration	*%zone_name%*:Zone Air CO2 Concentration [ppm](TimeStep)	ppm	%*zone_name%* = [PLENUM-1, PERIMETER_ZN_1, PERIMETER_ZN_2, PERIMETER_ZN_3, PERIMETER_ZN_4, CORE_ZN]

### The file structure of the dataset

The dataset generator produces a structured set of output CSV files, EnergyPlus input files, and weather data to ensure that simulations are repeatable and that all CSV dataset files are easily accessible through basic scripts. Fig. 1 illustrates the file structure and naming conventions used in the *‘Dataset_output/’* folder. The folder structure includes a unique identifier *(%city_name%*) based on the name of the weather file used, a user-chosen prefix identifier (*%prefix_identifier%*) to categorize different simulation runs, an identifier for simulation timestep intervals used (*%interval%*), and a simulation index (*%n%*) for the user-chosen setting ‘*number_of_stochastic_EP_cases_to_generate*', where*%n%* represents the index number for the same type of simulation run with the same*%prefix_identifier%*.

All the sub-folders with the same*%prefix_identifier%* will have the same simulation settings such as simulation timestep and enabled/disabled use of random HVAC mode/HVAC setpoints/window opening signals. It is important to note that all the sub-folders with different*%n%* (but the same*%prefix_identifier%*) will have different randomness, including input signals for HVAC mode, HVAC setpoints and window opening, and stochastic occupancy for the five zones. The final time-series dataset output from EnergyPlus is*%City_name%_%n%*_*output.csv*. The simulation can be re-run again from the '*temp*' folder where all the required input files for EnergyPlus are copied.

## Discussion and conclusion

The open-source tool, *synconn_build,* described in this paper enables the generation of synthetic time series datasets for building dynamics, which can be used to validate control-oriented data-driven models. The highly customizable working of *synconn_build* allows greater control over the quality of building dynamics data. As the number of control variables increases, the intricacies of the building dynamics become more complex, posing challenges for neural networks. Window openings and HVAC modes contribute to transient behavior and intricate thermal patterns that require advanced prediction methods. When these control variables undergo frequent changes, the intricacies intensify, compounding the prediction difficulty. However, through careful design of the signals and/or judicious selection of control variables, users can progressively amplify the complexity level of building dynamics while enhancing the prediction model. A progressive approach, starting with simpler models and fewer variables, can help in understanding the foundational dynamics. As familiarity and model performance improve, more complexity can be added iteratively. This layered approach not only aids in improving the prediction accuracy but also provides insights into how different variables impact overall building behavior.

The tool's output is a text-based CSV file that contains time profiles for all internal loads, weather data, indoor air temperature, CO_2_ concentration, and HVAC energy use. The user has complete control over the generation of the random signals for the control variables and the energy simulation boundary conditions, as well as the temporal granularity of the output data. *Synconn_build* can also be customized to instruct EnergyPlus to provide additional HVAC data (like supply temperatures, flow pressures and coil inlet/outlet temperatures). Additionally, users can customize their EnergyPlus IDF files with different building geometry and components in similar way shown the example 5-zone office building IDF template file.

Moreover, datasets generated by *synconn_build* allows for pre-training data-driven models with basic building dynamics learnings, following the transfer learning principles. The pre-trained models can be continuously tuned using collected operation datasets from real buildings, enabling more robust and adaptive HVAC control. The control variables selected for this tool (HVAC setpoints, HVAC mode, and window opening) may be sufficient for a good control algorithm, although additional variables could be included in future work. A similar methodology of creating a dataset using random signals can be applied to other control variables, expanding the scope of *synconn_build* 's application.

A stochastic data generator offers flexibility and efficiently captures various conditions, but it is not without challenges. A key concern is the authenticity of its randomness. If not genuine, it might create patterns unreflective of real-world situations. This randomness does not guarantee that all potential scenarios are represented, potentially overlooking crucial dynamics. Rare but vital scenarios could be overlooked, creating gaps in the understanding of the system dynamics. The randomness can also lead to bias or skewed data, with some scenarios over- or underrepresented. This lack of uniformity can mislead analytical evaluations.

Additionally, the generator may not capture the complex interdependencies present in real-world variables, leading to unlikely data combinations. Lastly, there is a risk of overfitting when training models on such data. When models are trained on data produced stochastically, there is a risk that they might fit too closely to the quirks and nuances of the generated data, rather than learning the broader patterns representative of real-world conditions. This can result in models that perform exceptionally well on the training data but fail to generalize effectively when exposed to new, unseen data.

## Code availability and usage

The data generator tool, *synconn_build,* is designed to be easily accessible and readily available for use. *Synconn_build*'s codebase is hosted on a public GitHub repository (https://github.com/gaurav306/synconn_build), and its V2.0 release [35] has been deposited on Zendo.org. To utilize *synconn_build*, users must have access to Python 3.10 and EnergyPlus v22.1.0. Both of these software packages are freely available for download and do not require a personal license for use. However, users should note that certain Python dependencies are required to run scripts of *synconn_build*, including *pandas*, PyYAML, *numpy, matplotlib, eppy, zipp, scipy*, and *statsmodels*. These dependencies can be installed using standard Python package management tools, such as pip. *Synconn_build* is licensed under the MIT license and is Windows operating system dependent.

**Fig. 15:**
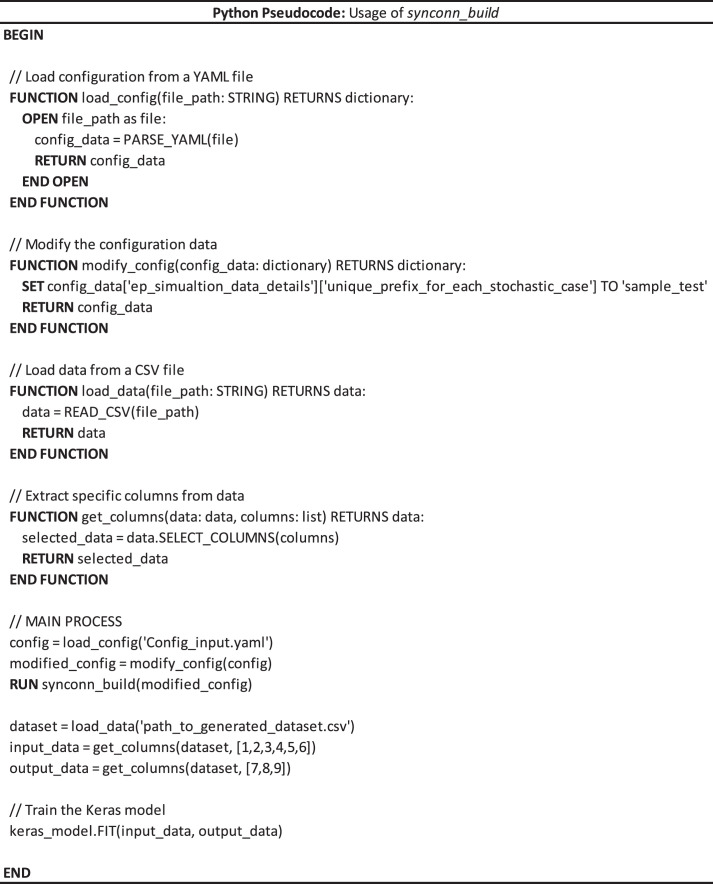
Pseudocode for usage of *synconn_build* and dataset generated.

To utilize this tool, the users would have to download the contents from GitHub's repository link. After inputting required settings in the *Config_input.yaml* file, the user can trigger data generation from the *Run_main.py* script in the GitHub codebase. Pseudocode for its usage and how data generated from it can be used for a black-box model is shown in Fig. 15.

## CRediT authorship contribution statement

**Gaurav Chaudhary:** Conceptualization, Methodology, Software, Writing – original draft. **Hicham Johra:** Visualization, Writing – review & editing, Supervision. **Laurent Georges:** Visualization, Writing – review & editing, Supervision. **Bjørn Austbø:** Visualization, Writing – review & editing, Supervision.

## Declaration of Competing Interest

The authors declare that they have no known competing financial interests or personal relationships that could have appeared to influence the work reported in this paper.

## Data Availability

No data was used for the research described in the article.

## References

[1] 2020 Global Status Report for Buildings and Construction: Towards a zero-emissions, efficient and resilient buildings and construction sector - executive summary 2020.

[2] Li H.S., Geng Y.C., Shinwari R., Yangjie W., Rjoub H. (2021). Does renewable energy electricity and economic complexity index help to achieve carbon neutrality target of top exporting countries?. J. Environ. Manag..

[3] Neukomm M., Nubbe V., Fares R. Grid-Interactive Efficient Buildings. United States: 2019. doi:10.2172/1508212.

[4] Mata É., Peñaloza D., Sandkvist F., Nyberg T. (2021). What is stopping low-carbon buildings? A global review of enablers and barriers. Energy Res. Soc. Sci..

[5] Drgoňa J., Arroyo J., Cupeiro Figueroa I., Blum D., Arendt K., Kim D. (2020). All you need to know about model predictive control for buildings. Annu. Rev. Control..

[6] Chen Y., Tong Z., Zheng Y., Samuelson H., Norford L. (2020). Transfer learning with deep neural networks for model predictive control of HVAC and natural ventilation in smart buildings. J. Clean. Prod..

[7] Yu X., Georges L., Imsland L. (2021). Data pre-processing and optimization techniques for stochastic and deterministic low-order grey-box models of residential buildings. Energy Build..

[8] Johra H., Schaffer M., Chaudhary G., Kazmi H.S., Le Dréau J., Petersen S (2023). What metrics does the building energy performance community use to compare dynamic models?. IBPSA.

[9] Pinto G., Messina R., Li H., Hong T., Piscitelli M.S., Capozzoli A. (2022). Sharing is caring: an extensive analysis of parameter-based transfer learning for the prediction of building thermal dynamics. Energy Build..

[10] Sartori I., Walnum H.T., Skeie K.S., Georges L., Knudsen M.D., Bacher P. (2023). Sub-hourly measurement datasets from 6 real buildings: energy use and indoor climate. Data Brief.

[11] Pipattanasomporn M., Chitalia G., Songsiri J., Aswakul C., Pora W., Suwankawin S. (2020). CU-BEMS, smart building electricity consumption and indoor environmental sensor datasets. Sci. Data.

[12] Ibarra A.M., González-Vidal A., Skarmeta A. (2023). PLEIAData: consumption, HVAC, temperature, weather and motion sensor data for smart buildings applications. Sci. Data.

[13] Miller C., Kathirgamanathan A., Picchetti B., Arjunan P., Park J., Nagy Z. (2020). The building data genome project 2, energy meter data from the ASHRAE great energy predictor III competition. Sci. Data.

[14] Li H., Wang Z., Hong T. (2021). A synthetic building operation dataset. Sci. Data.

[15] Hong T., Macumber D., Li H., Fleming K., Wang Z. (2020). Generation and representation of synthetic smart meter data. Build. Simul..

[16] Roth J., Martin A., Miller C., SynCity Jain R. (2020). Using open data to create a synthetic city of hourly building energy estimates by integrating data-driven and physics-based methods. Appl. Energy.

[17] Klemenjak C., Kovatsch C., Herold M., Elmenreich W. (2020). A synthetic energy dataset for non-intrusive load monitoring in households. Sci. Data.

[18] Knight W. (2016). Self-driving cars can learn a lot by playing grand theft auto. MIT Technol. Review.

[19] Shrivastava A., Pfister T., Tuzel O., Susskind J., Wang W., Webb R. (2017). Proceedings of the IEEE conference on computer vision and pattern recognition.

[20] Nikolaev E., Dvoryaninov P., Lensky Y., Drozdovsky N. (2018).

[21] Dahmen J., Cook D. (2019). SynSys: a synthetic data generation system for healthcare applications. Sensors.

[22] Chaudhary G., Johra H., Georges L., Austbø B. Predicting the performance of hybrid ventilation in buildings using a multivariate attention-based biLSTM encoder-decoder neural network. ArXiv Preprint ArXiv:230204126 2023.

[23] Chen Y., Hong T., Luo X. (2018).

[24] Althnian A., AlSaeed D., Al-Baity H., Samha A., Dris A.B., Alzakari N. (2021). Impact of dataset size on classification performance: an empirical evaluation in the medical domain. Appl. Sci..

[25] Reed R., MarksII R.J. (1999).

[26] Bishop C.M. (1995). Training with noise is equivalent to Tikhonov regularization. Neural Comput..

[27] Reynders G., Diriken J., Saelens D. (2014). Quality of grey-box models and identified parameters as function of the accuracy of input and observation signals. Energy Build..

[28] Crawley D.B., Lawrie L.K., Winkelmann F.C., Buhl W.F., Huang Y.J., Pedersen C.O. (2001). EnergyPlus: creating a new-generation building energy simulation program. Energy Build..

[29] Deru M., Field K., Studer D., Benne K., Griffith B., Torcellini P., et al. US department of energy commercial reference building models of the national building stock 2011.

[30] Winkelmann F.C. (2001). Modeling windows in EnergyPlus. Build. Simul..

[31] Torregrosa-Jaime B., Martínez P.J., González B., Payá-Ballester G. (2018). Modelling of a variable refrigerant flow system in EnergyPlus for building energy simulation in an open building information modelling environment. Energies.

[32] Wang C., Yan D., Jiang Y. (2011). A novel approach for building occupancy simulation. Build. Simul..

[33] Mahdavi A., Tahmasebi F., Kayalar M. (2016). Prediction of plug loads in office buildings: simplified and probabilistic methods. Energy Build..

[34] O'neill M.E (2014). PCG: a family of simple fast space-efficient statistically good algorithms for random number generation. ACM Trans. Math. Softw..

[35] Chaudhary G. Gaurav306/*synconn_build: synconn_build* 2023. doi:10.5281/zenodo.8121475.

